# Moving in extreme environments: open water swimming in cold and warm water

**DOI:** 10.1186/2046-7648-3-12

**Published:** 2014-06-11

**Authors:** Michael Tipton, Carl Bradford

**Affiliations:** 1 Extreme Environments Laboratory, Department of Sport and Exercise Science, University of Portsmouth, Portsmouth PO1 2ER, UK; 2 School of Physical Education, Sport and Exercise Sciences, University of Otago, Dunedin 9016, New Zealand

**Keywords:** Extreme environments, Open water swimming, Cold and warm water, Hyperthermia, Hypothermia, Acclimation, Acclimatisation, Exercise, Perception

## Abstract

Open water swimming (OWS), either ‘wild’ such as river swimming or competitive, is a fast growing pastime as well as a part of events such as triathlons. Little evidence is available on which to base high and low water temperature limits. Also, due to factors such as acclimatisation, which disassociates thermal sensation and comfort from thermal state, individuals cannot be left to monitor their own physical condition during swims. Deaths have occurred during OWS; these have been due to not only thermal responses but also cardiac problems. This paper, which is part of a series on ‘Moving in Extreme Environments’, briefly reviews current understanding in pertinent topics associated with OWS. Guidelines are presented for the organisation of open water events to minimise risk, and it is concluded that more information on the responses to immersion in cold and warm water, the causes of the individual variation in these responses and the precursors to the cardiac events that appear to be the primary cause of death in OWS events will help make this enjoyable sport even safer.

## Review

### Background

Open water swimming (OWS) includes competitive distances of between 5 and 25 km, including the first leg of triathlon events (e.g. up to Ironman, 3.8 km). OWS became an Olympic event in 2008; this acted as the stimulus for growth in a number of international competitive events taking place in a wide range of environmental conditions. In terms of numbers, perhaps even more importantly, OWS has caught the imagination of many thousands of people worldwide who now regularly engage in ‘wild swimming’ in rivers and remote waterways, or in open water mass participation events. These events are amongst the fastest growing mass participation sports worldwide with up to 25,000 participants entering single events.

With the increase in the number and variety of OWS events comes an increased risk of adverse medical events; both competitive and leisure swimmers have died in swimming events in warm and cold water. However, these deaths should be considered in the context of the potential improvements in health and longevity experienced by those who engage in such physical pursuits. This comparison is seldom made as the perception of risk has a temporal component, with acute risk (entering extreme environments) appearing much more hazardous than chronic risk (sedentary lifestyle).

Attempts to determine safety guidelines for swimming in open water have mostly focussed on thermal responses, but even in this limited area, such efforts have served to underline how difficult it is to prescribe limits when so many variables may interact, and the threshold between ‘safe’ and ‘unsafe’ may be very small. Obvious sources of variability in the thermal response include water temperature, metabolic heat production and insulation worn. More subtle causes include skill level, body morphology, wet suit ‘fit’, acute asymptomatic infection and radiant heat load. In addition to these considerations, others should be considered: the USA Triathlon Fatality Incidents Study [1] reported that 79% of deaths in triathlons in the USA between 2003 and 2011 occurred during the swim, with unexplained sudden cardiac death, rather than hypothermia or hyperthermia, being the most likely cause of death in most cases.

Thus, the evidence indicates that an open water swim represents the greatest relative hazard associated with mass participation sports events, and those searching for the cause of this hazard and ways of reducing it should look beyond just thermal responses. This paper, which is part of a series on ‘Moving in Extreme Environments’, briefly reviews pertinent topics associated with OWS.

A review of the relevant literature was undertaken. This involved the use of existing literature and bibliographies as well as relevant search engines where the keywords included are as follows: ‘cold water swimming’, ‘warm water swimming’, ‘open water swimming’, ‘FINA (the international governing body for aquatic sports) Regulations’, ‘ITU (International Triathlon Union) Regulations’, ‘IOC (International Olympic Committee) Regulations’, ‘British Triathlon Federation Regulations’, ‘cold and warm adaptation, acclimation, acclimatisation’, ‘thermal perceptions’, ‘drowning’, ‘hypothermia’ and ‘hyperthermia’. The resulting literature was critically reviewed and included in this review if hypotheses were tested, a clear methodology was described, appropriate analyses were undertaken and conclusions were supported by that analysis.

### Relevant regulations

**Figure 1:**
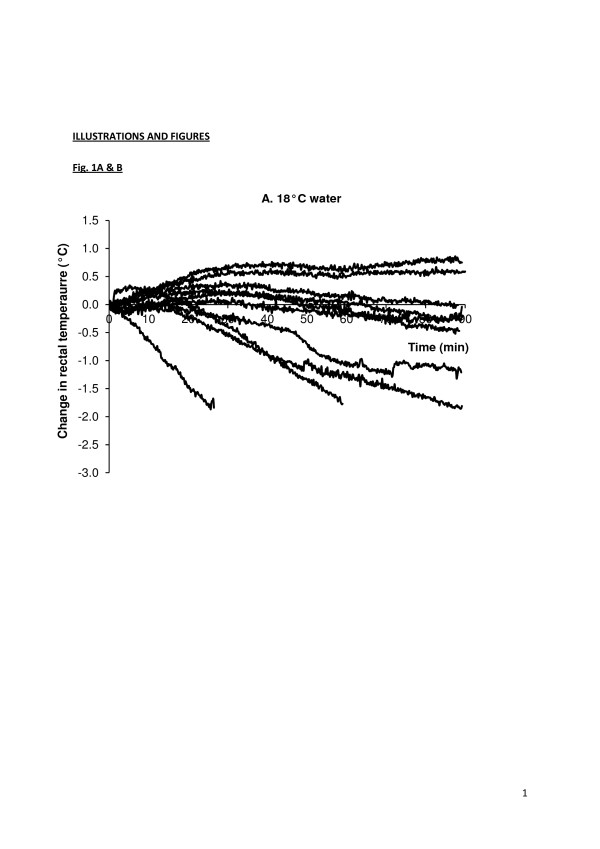
**Individual deep (rectal) body temperatures for individuals swimming at similar speeds in a swimming flume.** Water at **(A)** 18°C and **(B)** 10°C. Some individuals achieve thermal balance in both water temperatures (M Tipton, unpublished data, associated publication [9]).

There is no internationally accepted definition of ‘cold water’ or ‘warm water’. For most unacclimatised individuals, ‘thermoneutral’ water temperature (in which people can remain at rest and maintain deep body temperature without shivering or sweating) is within the narrow range of 35°C–35.5°C [2]. The narrowness of this range, resulting from a combination of the physical characteristics of water and the relative ineffectiveness of the physiological effector responses of humans to counter heat gain and loss in water, is the reason why the threshold between safe and unsafe water temperatures is more critical in water than in air. A water temperature of 25°C is generally regarded as the point below which exercise will accelerate the rate of deep body temperature cooling compared to remaining still. However, the range of water temperatures at which even naked individuals can achieve thermal balance when swimming is so varied, due to factors such as work intensity (heat production) and body morphology, that it is very difficult to set a single average figure for any form of guidance. For example, Costill et al. [3] observed that well-conditioned, non-obese individuals could demonstrate a slight increase in deep body temperature after 20 min of high-intensity exercise (VˑO_2_ of 3 L.min^-1^) in water at 17°C. Average swimmers swimming at similar speeds in water at 18°C and 10°C show a wide range of deep body temperature responses, including the re-establishment of thermal balance after an initial fall in deep body temperature (Figure 1A,B).

Many agencies define cold water as a temperature between 10°C and 15°C. Historically, such decisions have been based only on a consideration of deep body temperature and the threat of hypothermia (deep body temperature below 35°C). This is despite the fact that the most dangerous responses to immersion in cold water (‘cold shock’; see below) peak between 10°C and 15°C in naked or lightly clad individuals [4]. Indeed, the pre-occupation with hypothermia, which arose from the Titanic disaster and was maintained by events in World War II, is still reflected in the guidelines, policies and protection produced for cold water immersion.

In the UK, British Triathlon has the following guidelines:

• The minimum water temperature at which wetsuits are optional is 14°C.

• At temperatures less than 11°C, it is recommended that no OWS takes place.

At the following temperatures, the maximum swim distances are obligatory:

13°C, 2,000 m

12°C, 1,000 m

11°C, 500 m

The use of wetsuits is **forbidden**/*mandatory* if the following combinations of distance and water temperature are attained:

<1,500 m, >**22**°C/*<14*°C

1,501–3,000 m, >**23**°C/*<15*°C

3,001–4,000 m, >**24**°C/*<16*°C

Although well intentioned, the evidence base for these regulations is limited and virtually non-existent when it comes to OWS in warm water. Recently, some underpinning research has been sponsored and conducted to investigate endurance swimming in warm water and, latterly, cold water, with FINA (Fédération Internationale de Natation, the international governing body for aquatic sports), the ITU (International Triathlon Union) and IOC (International Olympic Committee) sponsoring this work and introducing a guideline of 31°C as the upper water temperature limit for OWS. In warm water, there is the likelihood of solar radiant heat (electromagnetic energy from the sun in the wavelength of 400–750 nm) adding up to 1,000 W.m^-2^ to the overall thermal load.

### Hazards associated with OWS in cold and warm water

As with all environmental stressors, the threat associated with OWS in thermally stressful water ranges from deterioration in performance to life-threatening pathology. The threats to life associated with immersion in cold water include drowning, cardiac problems, hypothermia and cardiovascular problems on exiting the water. In warm water, the corresponding threats are hyperthermia and cardiovascular problems on exiting the water.

The cardiovascular problems on leaving the water are caused by reduced circulating blood volume in cold and warm water. In cold water, this is due to hydrostatic squeeze and cold-induced vasoconstriction, producing a diuresis during immersion. In warm water, it is due to hydrostatic squeeze and sweating, resulting in hypovolaemia and the demand for high skin blood flow during immersion. Both sets of responses can compromise the maintenance of arterial blood pressure when leaving the water and assuming an upright posture (loss of hydrostatic squeeze plus orthostatic stress). This problem may be compounded by the removal of a tight-fitting wet suit at this time.

#### Cold water

In cold water, the cold shock response on initial immersion [5] includes a gasp response, uncontrollable hyperventilation, tachycardia, hyperventilation and an increase in circulating levels of stress hormones. The response is initiated by the dynamic response of the peripheral cold receptors; it peaks in the first 30 s of immersion and adapts over the first 2 min. The loss of control of breathing on immersion can be a precursor to drowning. That most of the deaths during OWS are thought to be due to cardiac problems raises interesting questions concerning the mechanisms associated with these deaths and why they tend to occur in competition or events rather than open water training or non-competitive swimming. Recent work [6] has suggested that coincidental activation of the sympathetic and parasympathetic inputs to the heart (‘autonomic conflict’) may provoke cardiac arrhythmias which, in individuals with predisposing conditions, can descend into fatal arrhythmias. This is more likely to occur in competition or other mass participation events because these more often involve, in addition to immersion in cool/cold water and exercise, extended breath holding, aspiration of water into the nasopharynx and anger [7]. Of all the emotions, anger is the one most associated with ventricular fibrillation; it increases the sympathetic tone while maintaining parasympathetic tone [8,9]. Acceptance of this theory has some implications for the design of mass participation swimming events (see below).

Following skin cooling, the next tissues to be affected are the superficial nerves and muscle, particularly in the upper limbs [10-12] which have a high surface area to mass ratio. The contractile force of muscle is significantly impaired when its temperature falls below 27°C due to factors such as reduced enzyme activity, decreased acetylcholine and calcium release, slower rates of diffusion, decreased muscle perfusion, increased viscosity and slower rate of conduction and repolarisation of action potentials [13]. The deep muscles of the forearm can reach this temperature after about 20 min at rest in 12°C or 40 min in 20°C water [14]. Below *T*_local_ of 20°C, rate of conduction and amplitude of action potentials is slowed; for example, the conduction velocity of the ulnar nerve falls by 15 m.s^-1^ per 10°C fall in *T*_local_. As a consequence of these changes, maximum power output falls by about 3% per degree Celsius fall in muscle temperature [15,16].

These changes in physiological function as a result of cooling can result in early swim failure across the spectrum of novice to elite swimmers, with novice swimmers suffering the biggest decrements, probably due to a lack of acclimatisation (see below) and having a less entrained motor programme for swimming and therefore more vulnerable technique.

In addition to direct local effects, with more generalised muscle and deep body cooling, there is a decrease in limb blood flow; this has occurred by the time deep body temperature reaches 36°C [17]. It is probable that the exercise hyperaemia normally observed in thermoneutral or warm conditions is attenuated in cooled individuals by a sympathetically mediated vasoconstriction of muscle resistance vessels [17,18]. As a consequence, oxygen delivery to, and utilisation by, cooled working muscle and the removal of the end products of metabolism may be reduced in cold water. This is compounded by a left shift in the oxygen dissociation curve with cooling. Cooled muscle is therefore required to use anaerobic metabolism at lower sub-maximum workloads; this can result in an earlier appearance of blood lactate [19] and more rapid depletion of carbohydrate stores and, as a consequence, an earlier onset of fatigue [20,21].

**Figure 2:**
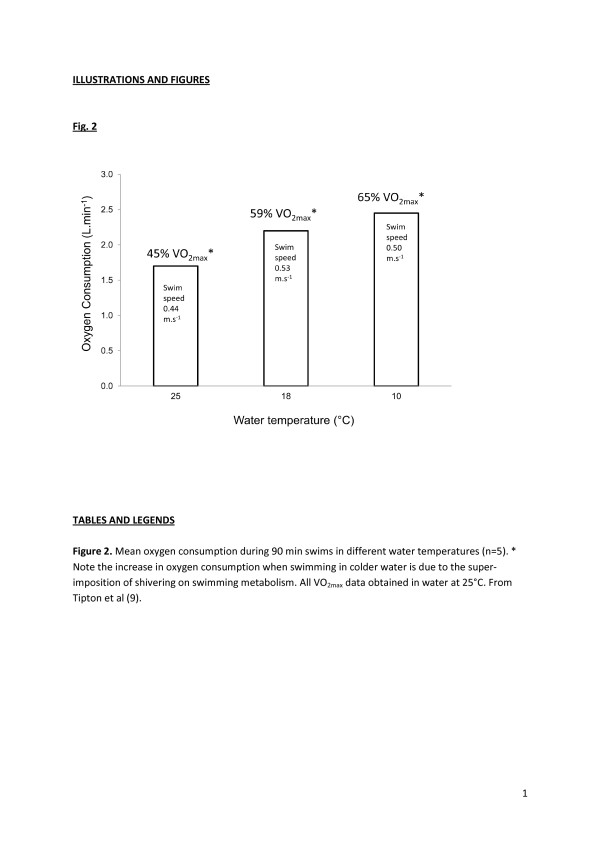
**Mean oxygen consumption during 90-min swims in different water temperatures (*n*** **= 5).** The increase in oxygen consumption when swimming in colder water is due to the super-imposition of shivering on swimming metabolism. The *asterisk* indicates that All VO_2max_ data were obtained in water at 25°C (from Tipton et al. [9]).

Higher oxygen consumptions have been noted in cold compared with neutral environments during exercise requiring oxygen consumptions of up to 2.0 L.min^-1^[11,19,22,23] (Figure 2), but not at 3.0 L.min^-1^[3]. In water at 25°C and 18°C, average oxygen consumption during arm and leg ergometry is increased by 9% and 25.3%, respectively, when compared to that seen in water at 33°C. The increase in oxygen consumption is greater in leaner individuals [24]. The twofold increase in the viscosity of water at 0°C compared with 25°C may contribute to the greater energy expenditure seen during swimming in cold water. However, this effect is compensated to some extent by the increased pulling power per stroke which results from the increase in viscosity. Although the efficiency of each stroke may improve, the shivering and increased muscle tone during exercise in cold water may further reduce the mechanical efficiency by increasing the activity of antagonistic muscles [25].

Maximum aerobic capacity falls in relation to muscle and deep body temperature, with a 0.5°C fall in deep body temperature resulting in a 10%–30% fall in VˑO_2_ max and maximum cardiac output (*Qˑ*_max_). In water at 18°C, the subjective sensations associated with exhaustive swimming are related to muscle function rather than cardio-respiratory distress [10].

There is little evidence to indicate that the alterations associated with cold water immersion impair respiratory function to an extent that it interferes with oxygen uptake during exercise [26]. With regard to cardiac function, once the cold shock response has subsided, cold water immersion reduces resting, submaximal and maximal heart rates when compared with those seen in warm water [27], with cardiac output being maintained during submaximal exercise in cold water by an elevated stroke volume [24]. The central redistribution of blood volume on immersion in cold water, caused by peripheral vasoconstriction and hydrostatic pressure, results in a cold-induced diuresis that can reduce circulating plasma volume by 24% [21,28]. This can further reduce muscle perfusion and, as mentioned, cause problems on exiting the water.

During resting immersions in cold water, a conductive gradient between the deep body tissues and the skin is established down which heat flows. In this situation, the deep body tissues always have a higher temperature than deep and superficial muscles which remain at a higher temperature than the skin. In contrast, during swimming, the exercise-induced hyperaemia destroys the ‘variable’ insulation provided by unperfused muscle (providing approximately 70% of total body insulation) when at rest, leaving only the ‘fixed’ resistance of subcutaneous fat. This may be especially relevant in swimming where, combined with other factors such as the surface area to mass ratio, exercise in water that involves the arms can lead to greater heat losses and faster decreases in deep body temperature compared to leg-only exercise at the same intensity [29]. Additionally, and importantly, the thermal mixing that occurs, due to exercise, between the deep tissues and the exercising muscle means that these tissues have a much more uniform temperature. The major consequence of this is that it is possible for swimmers to swim to the point of unconsciousness because this occurs, on average, at a deep body temperature of 30°C–33°C, whereas muscle function is maintained down to a temperature of about 27°C. This has been reported anecdotally (Phil Rush, personal communication). Also, in 1953, Jason Zirganos (JZ), the greatest open water swimmer of his generation, swam in the Bosphorus (8°C) for 4 h; he was removed from the water semiconscious, regaining full consciousness 3 h later. Unaware of hypothermia, it was concluded that he had been poisoned. The following year, at the age of 46 years, JZ attempted to swim the 22-mile North Channel of the Irish Sea (9.4°C–11.7°C). After 6 h, and only 3 miles from the Scottish Coast, JZ became unconscious and blue; he was hauled from the water, and a doctor, using a pen knife, exposed JZ's heart to reveal ventricular fibrillation. Direct cardiac massage having failed, JZ was pronounced dead at the scene (Griff Pugh, personal communication to M Tipton, 1982).

At the end of a cold water swim and for a period after it, the deep body temperature of a swimmer may continue to fall due to thermal gradients established during the swim [30]. Thus, this post-immersion period deserves attention in terms of the supervision of swimmers who, on finishing their race, may have the lowest deep body temperature they have experienced whilst unsupervised and travelling home, or, in the case of triathletes, go from swimming to cycling on a cold, wet day, and performance may be significantly impaired. Although the area of post-cold immersion rewarming has been well reviewed in the survival-related literature [30-32], it is less well considered in the sporting literature [33].

One final word on the oft quoted anecdotal benefits of cold OWS is that those who engage in this pastime claim health benefits ranging from improved immunity to greater alertness. There is no doubt that immersion in cold water stimulates release of the stress hormones even in habituated individuals, although to a lesser extent [34]. The alerting and arousal effect of cold immersion is likely to be one consequence of this. As for improved immunity, Jansky et al. [35] have reported that a single, 1-h immersion in 14°C which increased metabolic rate due to shivering activated the immune system to a slight extent. Brenner et al. [36] have confirmed that cold exposure (5°C air, 2 h) can be immune-stimulating, possibly due to increased levels of circulating noradrenaline. Having reviewed the area, Castellani et al. [37] concluded that there was no evidence to suggest that *moderate* acute cold, wet exposures depress the components responsible for immune function. In contrast, Shephard and Shek's [38] review concludes that the effect of *severe* chilling of mainly small mammals results in the suppression of several cellular and humoral components of the immune response, including a decrease of lymphocyte proliferation, a downregulation of the immune cascade and a reduction of natural killer (NK) cell count. Interestingly, adaptation of these responses to a given cold stimulus appears to develop over the course of 2–3 weeks. Regular short-duration cold water immersions or swimming have been associated with a ‘hardening’ response to oxidative stress and a consequent protective effect against free radical-induced tissue damage [39].

With regard to repeated cold water swimming, we await the definitive study on the interaction between cold exposure, exercise and the immune response in humans. To be definitive, this topic should be studied in a matched group of regular indoor swimmers to isolate the changes produced by regular swimming *per se* from those of swimming in cold water.

#### Warm water

The effector responses of the human thermoregulatory system evolved to function in thermoneutral dry air (26°C–28°C) in which sweat evaporation and cutaneous vasodilatation are efficient effector responses for off-loading heat from the body to the environment. When swimming in warm water the negation of the primary effector response for cooling, the evaporation of sweat, can be compensated for by the fact that the body is immersed in a fluid with much better physical characteristics for removing heat^a^. However, as the skin temperature-water temperature gradient narrows, less and less heat can be transferred to the water. If water temperature exceeds skin temperature, cutaneous vasodilatation picks up heat from the skin and returns it to the deep body tissues. This reversal of the normal function of cutaneous vasodilatation results in a rapid rise in deep body temperature. In contrast, on immersion in cold water, the physiological responses and morphological characteristics which evolved to keep individuals warm in cold air (shivering, peripheral vasoconstriction, subcutaneous fat) also function in cold water. So, it takes approximately *five times* longer to reduce the deep body temperature of someone in 15°C water (22°C below deep body temperature) from 37°C to 25°C (average lower lethal deep body temperature) than it does to raise their body temperature from 37°C to 40°C when they are immersed in 41°C water (just 4°C above deep body temperature). This is a powerful example of the impact of having the physiology of the body working for, as opposed to, against you. Given that the average lethal upper deep body temperature is 44°C, the rate at which this can be approached has been a cause for concern in warm water swimming events.

Little is known about the physiological responses to high-intensity endurance swimming in warm water, even though its popularity is increasing. Many 5–10-km events, which require athletes to be in the water for up to 2 h or more, are being held in locations such as the Middle East and South China Sea where water temperatures are up to 32°C. At rest, such temperatures represent a comfortable aquatic environment for humans, a little below the thermoneutral range. However, the effects of exercising at high metabolic rates in these conditions on thermoregulation (both behavioural and autonomic) are largely unknown, but the increase in metabolic heat production coupled with perceptions of comfort in these warm water environments appears to have the potential to induce ‘insidious hyperthermia’ in exercising athletes [40].

Only a few studies have examined intense or prolonged exercise in water, and even fewer have looked specifically at swimming. Of these studies, the use of different swimming strokes, relatively low and controlled exercise intensities (e.g. 50% VˑO_2_ max or lower) and short exercise durations (e.g. 30 min or less) make it difficult to apply the results to competitive race swimming, particularly of prolonged duration and in open water environments [10,19]. Also, many of these studies have been conducted in a swimming pool as opposed to a swimming flume, which allows for continuous swimming that is more representative of OWS. Robinson and Somers [41] conducted one of the few studies that have used proficient swimmers, in warm water temperatures, with exercise intensities and durations appropriate to competitive endurance swimming events. They asked six male Olympic-level swimmers to swim as far as possible in 60 min in three different water temperatures averaging 33.5°C, 29°C and 21°C. The rectal temperatures of the two fastest swimmers both increased to a modest 38.4°C after 60 min in the 33.5°C water (swimming at a metabolic rate of 500–520 kcal.m^-2^.min^-1^). One of the only other studies [42] had competitive Masters swimmers complete a 5-km race simulation in three water temperatures of 23°C, 27°C and 32°C. Rectal temperature (measured using mercury thermometers before and after each swim) showed a rise of 1.1°C in 32°C water. The peak rectal temperatures recorded after the 5-km swims in 27°C and 32°C water (which took on average 75–80 min) were only approximately 38°C. While there may be issues with the measurement methods, these recorded temperatures after 75 min of swimming at high intensity in 32°C water are not excessively high.

Thus, while there is some research examining the physiological effects of swimming in warm water, methodological limitations such as the use of pools, relatively short exercise times, discontinuous measurement of some physiological variables and a lack of radiant heat load make the application of these results to longer duration open water swims difficult. This may be important as it is immersion and exercise in warm (and cold) water for extended periods (i.e. greater than 30 min) that can lead to potentially dangerous deep body temperatures in swimmers. There is also a lack of research examining whether individuals can accurately perceive these changes in deep body temperature and initiate appropriate behavioural thermoregulatory responses when swimming in water.

Recent data [43] indicate that simulated OWS (conducted in a flume, at race pace and with radiant heat loading) by competitive swimmers and triathletes in 32°C water for 20, 60 or 120 min elicits modest increases in rectal temperature (mean [SD] 38.1°C [0.4], 38.3°C [0.6] and 38.4°C [0.8], respectively). The highest temperature recorded in any individual swimmer was 39.5°C, and less than 10% of swims ended in rectal temperatures over 39°C. All swims were also associated with appropriate, linear increases in psychophysical measures of thermal sensation and thermal (dis)comfort (i.e. feel hotter and more uncomfortable as rectal temperature increases), and a negative relationship with overall feeling (i.e. feel worse as rectal temperature increases). Further, when compared to terrestrial-based exercise at similar skin temperatures, these swimmers appear to feel hotter and more uncomfortable at the same deep body temperature. These data support the previous conclusion that only modest increases in rectal temperature occur when swimming in these warm water temperatures. They also show that intense endurance exercise in a seemingly heat stressful aquatic environment may not be that ‘extreme’ or insidious and that swimmers are able to perceive increases in their thermal status, even with a ‘comfortably’ clamped skin temperature (see next section).

### Pros and cons of self vs. prescribed acute exposure

Despite the findings reported above [43], the fact that we see cases such as the death of Jason Zirganos or people in warm air and water who appear able to continue exercising to death, particularly in competitive scenarios, suggests that for some individuals, the average findings of others do not apply. It is not possible to determine if this is because these individuals are able to override or ignore thermally initiated drives to stop or whether for these individuals, these drives are absent. Either way, that this can happen means it is unwise to rely on the subjective assessment of some individuals to determine exposure time during OWS.

Normally, the assessment of one's thermal state is driven by skin temperature, particularly changes in skin temperature [44]. However, water clamps skin temperature, allowing the cutaneous thermoreceptors to adapt to their local temperature and thereby reduce their input to the perceptual and therefore behavioural response to immersion. This situation is observed on resting immersions in cold water where after an initial and profound reduction, thermal comfort and thermal sensation improve over the next minutes as skin temperature plateaus at a new lower temperature with a consequent withdrawal of the dynamic response of the cutaneous cold receptors and their adaptation to the new static temperature. Although deep body temperature can influence the perception of the thermal state of the body as well as the drive to exercise [45], it can be fooled; for example, ‘insidious hypothermia’—the undefended fall in deep body temperature—occurs when this temperature falls too slowly to evoke a defensive reaction [46].

Finally, and as discussed in the next section, acclimation or acclimatisation to an environment can dissociate the thermal state of the body from the evoked subjective perceptions. This makes subjective assessment a particularly unreliable indicator of exposure time and physical capability.

### Adaptation

**Figure 3:**
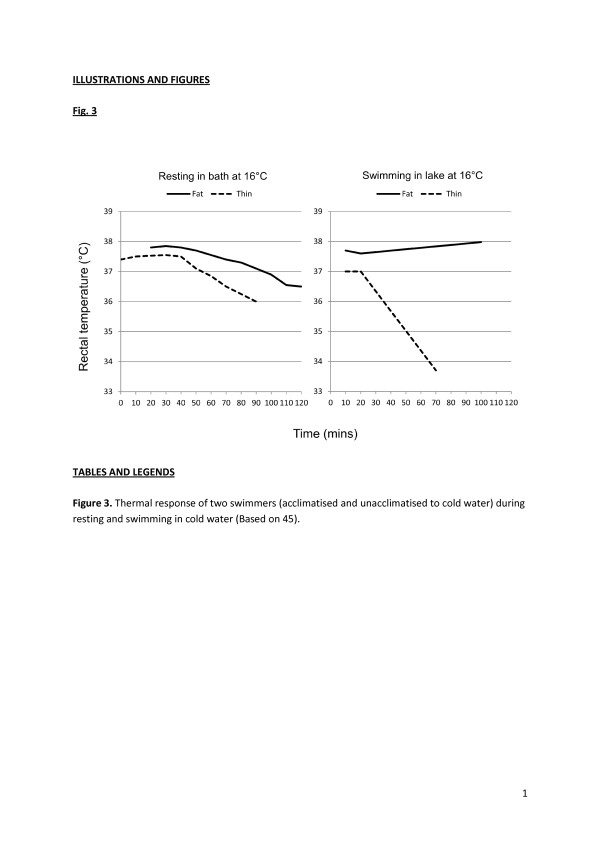
**Thermal response of two swimmers.** The swimmers were acclimatised and unacclimatised to cold water (based on [45]).

Whilst acclimation and acclimatisation to heat are well-researched and recognised processes, cold acclimatisation has been a more controversial topic. However, a fairly consistent pattern has emerged when those who repeatedly immersed themselves in cold water have been examined. In an attempt to determine why cross-channel swimmers could survive significantly longer in cold water than shipwreck victims from the Second World War, Pugh and Edholm [47] undertook early controlled studies of these swimmers. They concluded that the swimmers had a somewhat unique combination of fatness and fitness that allowed them to maintain a high level of heat production and retain it below significant levels of insulation. Actually, the best evidence for acclimatisation to cold was seen during resting immersions in which the outdoor swimmer (JZ) demonstrated an almost completely habituated shivering response, high levels of thermal comfort but a much faster fall in rectal temperature than would be expected in someone with this morphology; this response has been called ‘hypothermic’ acclimatisation (Figure 3). The high levels of thermal comfort despite low and faster falling deep body temperatures essentially represent the disabling of the behavioural thermoregulatory system [48] and add to the argument that regular outdoor swimmers (particularly in the cold) should not be allowed to self-regulate their exposures.

Some years later, Golden et al. [49] showed that outdoor swimmers could be thin provided they were fast, they could be fat and fast, and they could be fat and slow; what they could not be was thin and slow! Golden et al. [49] claimed evidence of insulative acclimatisation to cold during swimming in cold water. This claim has received recent support from studies of both adult and child open cold water swimmers [50,51].

Thus, it seems that the current best indications are that cold water swimmers develop a hypothermic acclimatisation to resting immersions [47,48]. Recent evidence indicates that this acclimatisation is limited to the thermal profile experienced repeatedly, and an un-habituated response returns if an individual cools more than he or she is used to [52]. During swimming cold water immersions, the acclimatised cold water swimmer demonstrates an insulative acclimatisation with greater levels of body insulation and better maintained deep body temperature [49,51].

There is a plethora of research examining the physiological and work capacity changes that occur with heat acclimation; however, almost all have been conducted in warm air and cannot be assumed to have much relevance to aquatic athletes or workers. Typical adaptations regularly observed with terrestrial heat acclimation include (i) a decrease in basal deep body temperature at rest and during exercise, (ii) an increase in sweat rate, (iii) an increase in blood volume and reduction in cardiovascular strain during exercise, (iv) improved thermotolerance and (v) improved perceptions (less exertion and thermal discomfort). These adaptations occur in response to a regular elevation in deep body temperature along with skin temperature and appear to be more complete if higher temperatures are achieved and there is an exercise component to the acclimation. Therefore, with the modest increases in deep body temperature that have been observed to date for exercise in warm water, the lack of heat-induced lowering of central venous pressure and skin temperature being clamped in warm water (i.e. 30°C–33°C), it is possible that these adaptations may not occur with heat acclimation in water, or if responses such as increased sweating power occur, they may even be counterproductive, producing faster dehydration, as has been shown for working in encapsulated ensembles [53]. However, research indicates that certain heat shock proteins may play a significant role in the thermotolerance afforded by heat acclimation. Further, it appears that these proteins may be induced both by duration (i.e. time for which deep body temperature is raised; the ‘dose’ of heat) and intensity (i.e. lower deep body temperature but a higher rate of increase) of exercise [54,55]. Thus, while the combination or level of stress imposed by repeated exercise in warm water may not result in the typical heat acclimation adaptations such as lowered basal deep body temperature and increased blood volume, it may be sufficient to provide improvements in thermotolerance.

The research is very limited with regard to heat acclimation in warm water. Weller et al. [56] used 4 days and Weller and Harrison [57] used 10 days of 30-min passive heating in 40°C water followed by 40-min cycling exercise in warm air, aiming to improve the performance of soldiers in hot environments wearing encapsulated clothing. While they observed the typical heat acclimation adaptations, it is impossible to differentiate the effects of the aquatic and terrestrial components to suggest whether hot water immersion alone would provide the adaptations.

Shin et al. [58] immersed nine males up to the waist for 30 min in 42°C water over a 3-week period (a total of ten immersions on alternate days). Although no heat stress test was conducted pre- and post-acclimation, they did observe a small but significant decrease in resting tympanic temperature (0.13°C) and an increase in whole-body sweat rate (estimated from body mass change) across the ten immersions. Lastly, Avellini et al. [59] took untrained participants and compared physical training (cycling at approximately 75% VˑO_2_ max) in water (32°C and 20°C) and on land (conditions not mentioned) for 1 h.day^-1^, 5 days.week^-1^, for 4 weeks to determine adaptations responsible for improving heat tolerance. Across the training sessions, rectal temperature increased approximately 1.1°C in the land-based exercise and approximately 0.6°C in the 32°C water-based exercise. Following the physical training period, a similar decrease in final deep body temperature and heart rate was observed at the end of a 3-h heat stress test (on land) between the 32°C water- and land-based training groups. Interestingly, the 32°C water training group also showed an increased sweat rate (by 25%) during the post-training heat stress test. Further, this physical training was followed by an actual 10-day heat acclimation period in warm air. The final deep body temperature recorded in a 3-h heat stress test after this acclimation period again fell by a similar amount in both the 32°C water- and land-based training groups. These data indicate that repeat exercise exposure in air and in 32°C water can provide similar training benefits and adaptive responses that improve heat tolerance on land. However, this study used untrained participants and upright cycling exercise, the physical training period (discussed here as acclimation) was 1 month long, and the heat stress tests used to examine heat tolerance were performed on land. Nonetheless, together with the previous studies, they do provide some evidence for the use of a warm water medium to successfully heat acclimate individuals. However, given the different responses seen with resting and exercising immersions in cold water, it is worth noting that no research appears to have specifically examined the acute and adaptive responses to repeated warm water exposures with swimming.

Recent research by one of the authors has started investigating this question with eight male competitive swimmers, in a randomised crossover study, completing a 7-day water-based heat acclimation (HA) and control (CONT) period. Acclimation involved 60 min of flume-based swimming in 33°C water with 20-min performance swims completed before and after HA and CONT. Rectal temperature rose approximately 1°C in most HA sessions. However, there were no clear differences between the pre- and post-performance swims in any of the typical adaptations regularly observed with terrestrial heat acclimations. For example, HA did not improve 20-min swim performance in warm or temperate water and did not lower resting or exercising rectal temperature or heart rate, sweat rate was not increased, and there was no consistent plasma volume expansion. Only thermal perceptions appeared to be improved, with swimmers feeling slightly cooler during performance in warm and temperate water. Thus, 7 days of flume swimming in uncomfortably hot water temperatures is minimally effective at inducing typically observed heat adaptations, possibly associated with the lack of orthostatic stress and limited hyperthermic strain (C Bradford, unpublished 2014).

### Summary and future directions

Problems during OWS can occur as a result of reductions in muscle temperature and reductions and increases in deep body temperature. There remains incomplete understanding of the responses to warm water swimming and the influence of acclimatisation, acquired from swimming in warm water, on these responses. The sources of the individual variation in the responses to cold and warm water exercising immersions are not fully understood. The mechanism that allows some people to override protective cues and exercise to the point of death remains a hazardous mystery.

Cardiac arrhythmias, with or without underlying pathology, may explain some of the deaths seen during OWS and are the prime suspect for those otherwise unexplained deaths that cannot be associated with thermal changes within the body or drowning. The new theory of autonomic conflict as a mechanism of sudden death on immersion and in other scenarios requires further study. Given that autonomic conflict is most likely when swimming in large groups and that the first part of a swim (up to 400 m) is where the greatest number of general incidents occur, it would seem sensible to adopt the general guidelines listed below when organising an OWS event. These guidelines are all designed to minimise the need for breath holding, the chance of aspirating water into the nasopharynx and the potential for crowding, conflicts and anger. The level of evidence underpinning these recommendations is weak/hypothetical; this is another area that requires further investigation.

1. Limit wave/group sizes.

2. Have a wide start line/course width, with the caveat that it can be properly surveyed.

3. Have reasonable time gaps between wave starts.

4. Have a good number of easily visible (from water level) buoys to prevent sharp turns.

5. At the start, have as long a straight line distance before requiring swimmers to make a turn, allowing the swimmers to spread out and find their own pace.

6. Ask swimmers to ‘self-select’ into waves of appropriate ability or ask weaker/novice swimmers to start at the back of a wave.

7. Advocate acclimatisation, anxiety reduction and anger management.

8. Increase the amount of safety cover in the first 400-m section and at turns. Brief swimmers to take their time at the start (particularly if a slower, less fit or a novice swimmer).

## Conclusions

OWS is an increasingly popular sport that takes place in water temperatures that can present an additional risk to those already inherent in the sport and mass participation in it. More information on the responses to immersion in cold and warm water, the causes of the individual variation in these responses and the precursors to the cardiac events that appear to be the primary cause of death in OWS events will help make this enjoyable sport even safer.

## Endnotes

^a^The volume-specific heat capacity is obtained by multiplying the specific heat of a substance by its density. It represents the amount of heat required to raise the temperature of a given volume of water by 1 K. At 37°C, the volume-specific heat capacity of water is 3,431 times that of air.

## Competing interests

The authors declare that they have no competing interests.

## Authors' contributions

MT produced the first draft of the chapter and helped edit it to the final form. CB contributed to the sections on warm water and helped with the final editing. Both authors read and approved the final manuscript.
